# While the Chatbot's Away, the Mice Will Play

**DOI:** 10.3389/fdgth.2021.617013

**Published:** 2021-02-02

**Authors:** Vincent Misrai, Dominique Pon, Hélène Charbonneau

**Affiliations:** ^1^Department of Urology, Clinique Pasteur, Toulouse, France; ^2^General-Manager of Clinique Pasteur, Toulouse, France; ^3^Head of Digital Health Transformation Strategy, French Minister for Solidarity and Health, Toulouse, France; ^4^Department of Anesthesia and Critical Care, Clinique Pasteur, Toulouse, France

**Keywords:** artificial intelligence, healthcare, digital assistants, electronic health record, voice assistance systems

The names Douglas Carl Engelbart and Joseph Weizenbaum probably sound unknown to most physicians worldwide, but these engineers are forever belonging to a small revolution in the development of human-computer interactions. In 1966, Weizenbaum created what is considered as the mother of the chatbots, a natural language processing computer program called ELIZA (1). Engelbart created in 1968 what became the simplest manual interface with machines and a tool commonly used in the tertiary sector of the economy: the computer mouse.

Decades later, the healthcare system has not escaped the global digitalization trend. Medical paper files have been replaced by the patient Electronic Health Record (EHR), and the keyboard-and-mouse interface has superseded the pen. Thus, health center infrastructures have been transformed into modern mousetraps.

Although the EHR offered undisputable benefits for patients, data collection became rapidly a clerical burden for the physicians across all medical specialties and reported in aftermath as factor of burnout (2). Physicians' time was already strained in a “hamster health care system” (3). Twenty years later, these issues have only worsened and might call Engelbart' legacy into question. The time spent to support care delivery with repetitive mouse clicks has been reported to constitute a large portion of the physicians' day (4) and to impair patient participation in medical care conversations (5). As an illustration of the current situation, physicians click a computer mouse more than 200 times and a keyboard more than 700 times (5), accounting for up to 16 min per patient, when using the EHR for chart review, documentation, and to place orders (4).

Undoubtedly, EHR has still a bright future, but its adoption does not rely on an always more intuitive web platform aiming to provide at a glance all of patient's medical information through a firework of pop-up windows. The right human-to-EHR interface should enable doctors to regain some time for their patients and make healthcare human again (6). The new generation of interface relies on artificial intelligence and the development of natural language processing (NLP). From mouse to mouth: shifting the paradigm with the help of vocal assistants (7). Using voice-recognition software to interact with EHRs instead of the traditional keyboard-and-mouse interface could lead to a 50% reduction in the time required to complete patient encounters (8).

The ability for a system to understand a voice in a conversation is inextricably nested within speaker diarization process. A technical wording for a precise task that is usually summarized in answering the question “who spoke when?”. Speaker diarization achieved different level of accuracy and diarization error rate (which is the sum of three sources of error: missed speech, false alarm speech and speaker error) has substantially improved over the past years from >20% to less than 10% with the most efficient systems (9).

Chatbots are an expanding market taking benefits of a broad adoption in the general population (10). From the patient's perspective, chatbots are now able to answer frequent asked medical questions and remotely improve adhesion to the therapeutics.

From the physician's perspective, chatbot current versions are not ready for primary use in clinical practice (11) but their prospects are clearly identified: to automate all scripted and repetitive administrative workflows, collect all of the patient's data and help to make to best clinical decision based on a million of patient's database with respect to the ethical policies.

To overcome potential ethical pitfalls, it is therefore essential that voice recordings (speech data) are adequately protected so that they cannot be misused. Specific privacy-preserving techniques are needed to deliver biometric information protection for speaker characterization applications (12).

The difficult time we are currently living in should compel us to change and to go beyond complaining about EHRs and the obsolete tools physicians are using to be interconnected with. The COVID-19 pandemic has unveiled healthcare weaknesses and stroke human resources. Among all the possible routes for the virus to be transmitted, some investigations have shown that the hospital environment frequently becomes contaminated when providing care for COVID-19 patients. Keyboards and mice, the most common hospital items that already appropriate physician time, create even more anxiety as they could carry the pathogen (13).

That being said, should the professional healthcare get rid of all computer mice for chatbots? Certainly not.

There is no place in medicine that concentrates such a high level of innovative technology in a square meter as the operating room (OR).

Hand-controlled non-autonomous *Da Vinci* robots have revolutionized the standard of care, but in surgery, it is widely acknowledged that a surgeon's results improve with experience, and the definition of the number of procedures a surgeon needs to complete before being able to perform a procedure independently with a reasonable outcome is a never ending matter of debate. Clicking could fill the gap in surgical expertise and shorten the learning curve (14). A surgeon can simply repeatedly click a button indicating where to place sutures, without the need for any particular skill (14).

Meanwhile the rise in self-guided surgical robots, surgeons already started revisiting the benefits of clicking a computer mouse and keyboard in the OR. Trackballs are becoming a new interface between patients and machines, allowing the surgeon to set and conduct a semi-autonomous robotic-executed procedure, making the task more reliable with a reproducible outcome (Figure 1) (15). Voice assistants have the potential to improve radically how physicians will interact with computers. Mice should no longer stand as the only human-to-EHR interface but have still a place where to strive in the upcoming years: the OR.

**Figure 1:**
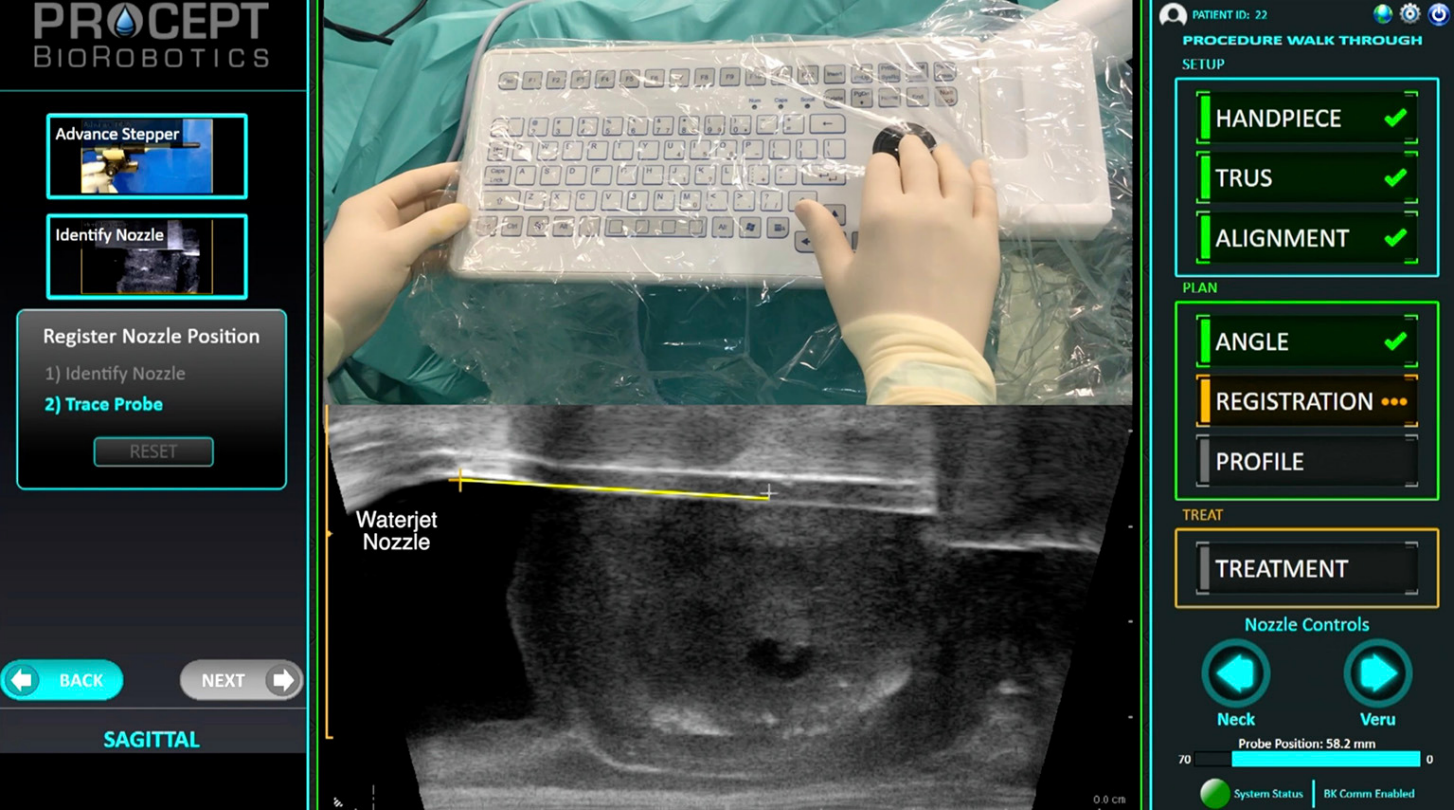
Robotic executed surgical treatment of benign prostatic hyperplasia with the Aquabeam system.

## Author Contributions

VM and DP wrote the manuscript. HC and VM revised the manuscript. All authors contributed to the article and approved the submitted version.

## Conflict of Interest

The authors declare that the research was conducted in the absence of any commercial or financial relationships that could be construed as a potential conflict of interest.
